# 6-[(*E*)-2-Phenyl­vin­yl]-1*H*-indole

**DOI:** 10.1107/S1600536811051750

**Published:** 2011-12-10

**Authors:** Yu-Hua Ge, Chen-Guang Zhang, Yang-Hui Luo

**Affiliations:** a Ordered Matter Science Research Center, College of Chemistry and Chemical Engineering, Southeast University, Nanjing 210096, People’s Republic of China

## Abstract

The title compound, C_16_H_13_N, is essentially planar [maximum deviation from the least-squares plane = 0.081 (3) Å], with a dihedral angle of 1.65 (13)° between the planes of the indole and benzene rings. In the crystal, there are no significant inter­molecular π–π inter­actions [minimum ring centroid–centroid separation = 4.217 (5) Å].

## Related literature

For background information on indole derivatives as drug inter­mediates, see: Kunzer & Wendt (2011 ▶).
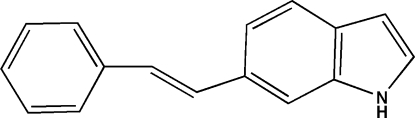


## Experimental

### 

#### Crystal data

C_16_H_13_N
*M* *_r_* = 219.29
Orthorhombic, 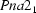

*a* = 8.254 (8) Å
*b* = 5.626 (6) Å
*c* = 25.74 (3) Å
*V* = 1195 (2) Å^3^
*Z* = 4
Mo *K*α radiation
μ = 0.07 mm^−1^
*T* = 296 K
0.30 × 0.20 × 0.10 mm

#### Data collection

Rigaku SCXmini CCD-detector diffractometer
Absorption correction: multi-scan (*CrystalClear*; Rigaku, 2005 ▶) *T*_min_ = 0.982, *T*_max_ = 0.993
7653 measured reflections
1954 independent reflections
1627 reflections with *I* > 2σ(*I*)
*R*_int_ = 0.022

#### Refinement

*R*[*F* ^2^ > 2σ(*F* ^2^)] = 0.046
*wR*(*F* ^2^) = 0.132
*S* = 1.08
1954 reflections
154 parameters
19 restraints
H-atom parameters constrained
Δρ_max_ = 0.27 e Å^−3^
Δρ_min_ = −0.21 e Å^−3^

### 

Data collection: *CrystalClear* (Rigaku, 2005 ▶); cell refinement: *CrystalClear*; data reduction: *CrystalClear*; program(s) used to solve structure: *SHELXS97* (Sheldrick, 2008 ▶); program(s) used to refine structure: *SHELXL97* (Sheldrick, 2008 ▶); molecular graphics: *DIAMOND* (Brandenburg & Putz, 2005 ▶); software used to prepare material for publication: *SHELXL97* and *PLATON* (Spek, 2009 ▶).

## Supplementary Material

Crystal structure: contains datablock(s) I, global. DOI: 10.1107/S1600536811051750/zs2163sup1.cif

Structure factors: contains datablock(s) I. DOI: 10.1107/S1600536811051750/zs2163Isup2.hkl

Supplementary material file. DOI: 10.1107/S1600536811051750/zs2163Isup3.cml

Additional supplementary materials:  crystallographic information; 3D view; checkCIF report

## Figures and Tables

**Fig. 1.**
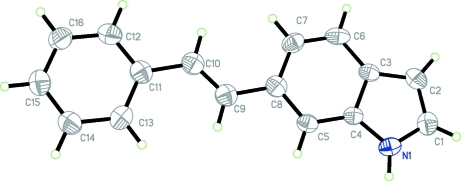
The molecular structure of the title compound with the atom-labelling scheme. Displacement ellipsoids are drawn at the 30% probability level. H atoms are represented as small spheres of arbitrary radii.

**Fig. 2.**
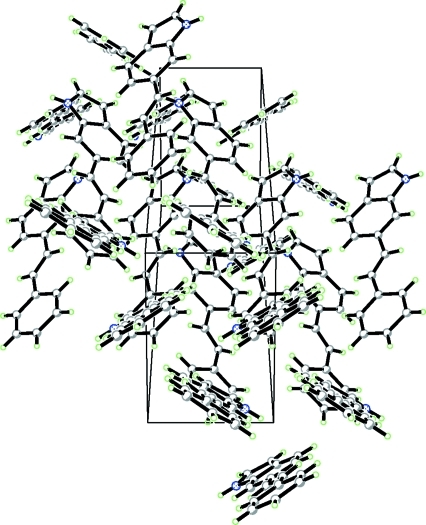
A packing in the unit cell viewed down the a axis.

## supplementary crystallographic information

### Comment

The derivatives of indole are important chemical materials, because they are excellent drug intermediates for many pharmaceutical products (Kunzer & Wendt, 2011). As part of our interest in these materials, we report here the crystal structure of the title compound C_16_H_13_N (Fig. 1).

This compound is essentially planar [maximum deviation from the least-squares plane 0.081 (3) Å (for C1)], with a dihedral angle of 1.65 (13)° between the planes of the indole and benzene ring systems. With the absence of no acceptor atoms in the molecule, no intermolecular hydrogen bonds are found in the crystal packing. Also, there are no significant intermolecular π–π interactions [minimum ring centroid separation, 4.217 (5) Å].

### Experimental

Crystals of 6-phenylvinylindole suitable for X-ray diffraction were obtained by slow evaporation of an ethanol solution.

### Refinement

All H atoms attached to C atoms and the N atom were fixed geometrically and treated as riding with C—H = 0.93 Å and N—H = 0.86 Å with *U*_iso_(H) = 1.2*U*_eq_(C or N).

### Crystal data

**Table:**

C_16_H_13_N	*F*(000) = 464
*M**_r_* = 219.29	*D*_x_ = 1.218 Mg m^−^^3^
Orthorhombic, *P**n**a*2_1_	Mo *K*α radiation, λ = 0.71073 Å
Hall symbol: P 2c -2n	Cell parameters from 1954 reflections
*a* = 8.254 (8) Å	θ = 3.2–25.0°
*b* = 5.626 (6) Å	µ = 0.07 mm^−^^1^
*c* = 25.74 (3) Å	*T* = 296 K
*V* = 1195 (2) Å^3^	Prism, colourless
*Z* = 4	0.30 × 0.20 × 0.10 mm

### Data collection

**Table:**

Rigaku SCXmini CCD-detector diffractometer	1954 independent reflections
Radiation source: fine-focus sealed tube	1627 reflections with *I* > 2σ(*I*)
graphite	*R*_int_ = 0.022
Detector resolution: 13.6612 pixels mm^-1^	θ_max_ = 25.0°, θ_min_ = 3.2°
CCD profile–fitting scans	*h* = −9→9
Absorption correction: multi-scan (*CrystalClear*; Rigaku, 2005)	*k* = −6→6
*T*_min_ = 0.982, *T*_max_ = 0.993	*l* = −27→30
7653 measured reflections	

### Refinement

**Table:**

Refinement on *F*^2^	Primary atom site location: structure-invariant direct methods
Least-squares matrix: full	Secondary atom site location: difference Fourier map
*R*[*F*^2^ > 2σ(*F*^2^)] = 0.046	Hydrogen site location: inferred from neighbouring sites
*wR*(*F*^2^) = 0.132	H-atom parameters constrained
*S* = 1.08	*w* = 1/[σ^2^(*F*_o_^2^) + (0.0665*P*)^2^ + 0.2586*P*] where *P* = (*F*_o_^2^ + 2*F*_c_^2^)/3
1954 reflections	(Δ/σ)_max_ < 0.001
154 parameters	Δρ_max_ = 0.27 e Å^−^^3^
19 restraints	Δρ_min_ = −0.21 e Å^−^^3^

### Special details

**Table:**

Geometry. All e.s.d.'s (except the e.s.d. in the dihedral angle between two l.s. planes) are estimated using the full covariance matrix. The cell e.s.d.'s are taken into account individually in the estimation of e.s.d.'s in distances, angles and torsion angles; correlations between e.s.d.'s in cell parameters are only used when they are defined by crystal symmetry. An approximate (isotropic) treatment of cell e.s.d.'s is used for estimating e.s.d.'s involving l.s. planes.
Refinement. Refinement of *F*^2^ against ALL reflections. The weighted *R*-factor *wR* and goodness of fit *S* are based on *F*^2^, conventional *R*-factors *R* are based on *F*, with *F* set to zero for negative *F*^2^. The threshold expression of *F*^2^ > σ(*F*^2^) is used only for calculating *R*-factors(gt) *etc*. and is not relevant to the choice of reflections for refinement. *R*-factors based on *F*^2^ are statistically about twice as large as those based on *F*, and *R*- factors based on ALL data will be even larger.

### Fractional atomic coordinates and isotropic or equivalent isotropic displacement parameters (Å^2^)

**Table:**

	*x*	*y*	*z*	*U*_iso_*/*U*_eq_	
C2	−0.0689 (4)	0.5222 (6)	0.31498 (14)	0.0656 (8)	
H2A	−0.0588	0.6242	0.2867	0.079*	
C8	0.0968 (4)	0.4941 (5)	0.46948 (12)	0.0597 (6)	
C7	0.1442 (3)	0.6940 (5)	0.43984 (13)	0.0623 (8)	
H7A	0.2102	0.8076	0.4553	0.075*	
C15	0.4035 (4)	0.4885 (6)	0.70717 (14)	0.0687 (9)	
H15A	0.4359	0.4764	0.7417	0.082*	
C5	−0.0018 (3)	0.3276 (5)	0.44626 (11)	0.0538 (7)	
H5A	−0.0369	0.1955	0.4648	0.065*	
C3	0.0004 (3)	0.5547 (5)	0.36510 (11)	0.0510 (6)	
N1	−0.1423 (3)	0.2156 (4)	0.36424 (10)	0.0594 (6)	
H1A	−0.1875	0.0850	0.3738	0.071*	
C6	0.0972 (3)	0.7280 (5)	0.38924 (13)	0.0613 (8)	
H6A	0.1289	0.8635	0.3712	0.074*	
C10	0.2538 (4)	0.5725 (6)	0.55023 (12)	0.0658 (5)	
H10A	0.3012	0.7004	0.5331	0.079*	
C4	−0.0482 (3)	0.3585 (4)	0.39512 (11)	0.0471 (6)	
C9	0.1498 (4)	0.4487 (6)	0.52418 (13)	0.0636 (5)	
H9A	0.1036	0.3191	0.5410	0.076*	
C12	0.4050 (4)	0.6954 (6)	0.62754 (15)	0.0720 (9)	
H12A	0.4403	0.8245	0.6080	0.086*	
C13	0.2544 (4)	0.3359 (6)	0.63444 (13)	0.0709 (9)	
H13A	0.1880	0.2199	0.6200	0.085*	
C11	0.3047 (4)	0.5306 (6)	0.60460 (13)	0.0640 (6)	
C14	0.3045 (4)	0.3175 (6)	0.68583 (14)	0.0716 (9)	
H14A	0.2710	0.1891	0.7059	0.086*	
C16	0.4541 (4)	0.6758 (7)	0.67764 (14)	0.0745 (9)	
H16A	0.5223	0.7901	0.6919	0.089*	
C1	−0.1528 (4)	0.3136 (6)	0.31610 (14)	0.0672 (8)	
H1B	−0.2087	0.2483	0.2882	0.081*	

### Atomic displacement parameters (Å^2^)

**Table:**

	*U*^11^	*U*^22^	*U*^33^	*U*^12^	*U*^13^	*U*^23^
C2	0.0589 (17)	0.074 (2)	0.0634 (19)	0.0063 (16)	0.0072 (14)	0.0118 (17)
C8	0.0534 (11)	0.0637 (12)	0.0620 (12)	0.0073 (10)	0.0089 (9)	−0.0038 (11)
C7	0.0517 (15)	0.0564 (17)	0.079 (2)	−0.0009 (13)	0.0019 (15)	−0.0132 (16)
C15	0.0648 (19)	0.078 (2)	0.063 (2)	0.0144 (17)	−0.0019 (16)	−0.0018 (18)
C5	0.0495 (14)	0.0506 (14)	0.0612 (19)	0.0012 (12)	0.0077 (13)	0.0047 (14)
C3	0.0395 (12)	0.0517 (14)	0.0618 (17)	0.0039 (11)	0.0095 (13)	0.0058 (13)
N1	0.0490 (12)	0.0554 (13)	0.0739 (18)	−0.0070 (10)	0.0006 (12)	−0.0002 (13)
C6	0.0507 (14)	0.0428 (13)	0.090 (2)	0.0006 (12)	0.0138 (16)	0.0090 (16)
C10	0.0604 (10)	0.0708 (11)	0.0661 (10)	0.0047 (9)	0.0093 (8)	−0.0033 (9)
C4	0.0389 (12)	0.0474 (13)	0.0550 (16)	0.0029 (10)	0.0045 (12)	0.0009 (12)
C9	0.0579 (9)	0.0675 (11)	0.0654 (10)	0.0049 (9)	0.0093 (8)	−0.0047 (9)
C12	0.0658 (19)	0.074 (2)	0.077 (2)	−0.0057 (16)	0.0101 (18)	0.0082 (19)
C13	0.0671 (19)	0.071 (2)	0.074 (2)	−0.0016 (16)	−0.0065 (18)	−0.0103 (18)
C11	0.0590 (11)	0.0707 (12)	0.0624 (12)	0.0082 (11)	0.0107 (10)	−0.0022 (11)
C14	0.073 (2)	0.070 (2)	0.072 (2)	0.0003 (16)	0.0026 (17)	0.0096 (17)
C16	0.067 (2)	0.078 (2)	0.078 (3)	−0.0089 (17)	−0.0027 (18)	−0.0035 (19)
C1	0.0569 (17)	0.082 (2)	0.0630 (19)	0.0074 (16)	−0.0034 (15)	−0.0041 (18)

### Geometric parameters (Å, °)

**Table:**

C2—C1	1.363 (5)	N1—C4	1.372 (4)
C2—C3	1.423 (5)	N1—H1A	0.8600
C2—H2A	0.9300	C6—H6A	0.9300
C8—C5	1.377 (4)	C10—C9	1.293 (4)
C8—C7	1.415 (4)	C10—C11	1.480 (5)
C8—C9	1.497 (5)	C10—H10A	0.9300
C7—C6	1.372 (5)	C9—H9A	0.9300
C7—H7A	0.9300	C12—C16	1.356 (5)
C15—C16	1.365 (5)	C12—C11	1.376 (5)
C15—C14	1.377 (5)	C12—H12A	0.9300
C15—H15A	0.9300	C13—C14	1.390 (5)
C5—C4	1.382 (4)	C13—C11	1.401 (4)
C5—H5A	0.9300	C13—H13A	0.9300
C3—C4	1.406 (4)	C14—H14A	0.9300
C3—C6	1.405 (4)	C16—H16A	0.9300
N1—C1	1.359 (4)	C1—H1B	0.9300
			
C1—C2—C3	107.2 (3)	C11—C10—H10A	116.8
C1—C2—H2A	126.4	N1—C4—C5	129.5 (2)
C3—C2—H2A	126.4	N1—C4—C3	107.7 (3)
C5—C8—C7	118.0 (3)	C5—C4—C3	122.9 (3)
C5—C8—C9	117.7 (3)	C10—C9—C8	126.2 (3)
C7—C8—C9	124.2 (3)	C10—C9—H9A	116.9
C6—C7—C8	123.0 (3)	C8—C9—H9A	116.9
C6—C7—H7A	118.5	C16—C12—C11	122.2 (3)
C8—C7—H7A	118.5	C16—C12—H12A	118.9
C16—C15—C14	119.9 (3)	C11—C12—H12A	118.9
C16—C15—H15A	120.0	C14—C13—C11	119.5 (3)
C14—C15—H15A	120.0	C14—C13—H13A	120.3
C8—C5—C4	119.5 (3)	C11—C13—H13A	120.3
C8—C5—H5A	120.3	C12—C11—C13	118.0 (3)
C4—C5—H5A	120.3	C12—C11—C10	118.0 (3)
C4—C3—C6	117.7 (3)	C13—C11—C10	124.0 (3)
C4—C3—C2	106.4 (3)	C15—C14—C13	120.3 (3)
C6—C3—C2	135.9 (3)	C15—C14—H14A	119.9
C1—N1—C4	109.1 (2)	C13—C14—H14A	119.9
C1—N1—H1A	125.5	C12—C16—C15	120.1 (3)
C4—N1—H1A	125.5	C12—C16—H16A	120.0
C7—C6—C3	118.9 (3)	C15—C16—H16A	120.0
C7—C6—H6A	120.5	N1—C1—C2	109.7 (3)
C3—C6—H6A	120.5	N1—C1—H1B	125.2
C9—C10—C11	126.4 (3)	C2—C1—H1B	125.2
C9—C10—H10A	116.8		
